# IL6/sIL6R regulates TNFα-inflammatory response in synovial fibroblasts through modulation of transcriptional and post-transcriptional mechanisms

**DOI:** 10.1186/s12860-020-00317-7

**Published:** 2020-10-30

**Authors:** Alvaro Valin, Manuel J. Del Rey, Cristina Municio, Alicia Usategui, Marina Romero, Jesús Fernández-Felipe, Juan D. Cañete, Francisco J. Blanco, Yolanda Ruano, Gabriel Criado, José L. Pablos

**Affiliations:** 1 Grupo de Enfermedades Inflamatorias y Autoinmunes, Instituto de Investigación Hospital 12 de Octubre (i+12), Madrid, Spain; 2 Present Address: Springer Healthcare Iberica SL, Madrid, Spain; 3 Unitat d’Artritis, Servei de Reumatologia, Hospital Clínic de Barcelona and Institut d’Investigacions Biomèdiques August Pí i Sunyer, Barcelona, Spain; 4 Laboratorio de Investigación Osteoarticular y del Envejecimiento, Instituto de Investigación Biomédica de A Coruña, INIBIC, A Coruña, Spain; 5 Servicio de Anatomía Patológica, Instituto de Investigación Hospital 12 de Octubre (i+12), Madrid, Spain; 6 Servicio de Reumatología, Hospital 12 de Octubre, Universidad Complutense de Madrid, 28041 Madrid, Spain

**Keywords:** IL6, TNFα, Soluble receptor, Synovial fibroblast, Inflammatory response, Rheumatoid arthritis, Cross-talk, Transcriptional mechanisms, Post-transcriptional mechanisms, JAK/STAT

## Abstract

**Introduction:**

The clinical efficacy of specific interleukin-6 inhibitors has confirmed the central role of IL6 in rheumatoid arthritis (RA). However the local role of IL6, in particular in synovial fibroblasts (SF) as a direct cellular target to IL6/sIL6R signal is not well characterized. The purpose of the study was to characterize the crosstalk between TNFα and IL6/sIL6R signaling to the effector pro-inflammatory response of SF.

**Methods:**

SF lines were stimulated with either TNFα, IL6/sIL6R, or both together, for the time and dose indicated for each experiment, and where indicated, cells were treated with inhibitors actinomycin D, adalimumab, ruxolitinib and cycloheximide. mRNA expression of cytokines, chemokines and matrix metalloproteases (MMPs) were analyzed by quantitative RT-PCR. Level of IL8/CXCL8 and CCL8 in culture supernatants was measured by ELISA. Mononuclear and polymorphonuclear cells migration assays were assessed by transwell using conditioned medium from SF cultures. Statistical analyses were performed as indicated in the corresponding figure legends and a *p*-value < 0.05 was considered statistically significant.

**Results:**

The stimulation of SF with IL6/sIL6R and TNFα, cooperatively promotes the expression of mono- and lymphocytic chemokines such as IL6, CCL8 and CCL2, as well as matrix degrading enzymes such as MMP1, while inhibiting the induction of central neutrophil chemokines such as IL8/CXCL8. These changes in the pattern of chemokines expression resulted in reduced polymorphonuclear (PMN) and increased mononuclear cells (MNC) chemoattraction by SF. Mechanistic analyses of the temporal expression of genes demonstrated that the cooperative regulation mediated by these two factors is mostly induced through de novo transcriptional mechanisms activated by IL6/sIL6R. Furthermore, we also demonstrate that TNFα and IL6/sIL6R cooperation is partially mediated by the expression of secondary factors signaling through JAK/STAT pathways.

**Conclusions:**

These results point out to a highly orchestrated response to IL6 in TNFα-induced SF and provide additional insights into the role of IL6/sIL6R in the context of RA, highlighting the contribution of IL6/sIL6R to the interplay of SF with other inflammatory cells.

**Supplementary information:**

**Supplementary information** accompanies this paper at 10.1186/s12860-020-00317-7.

## Background

Interleukin-6 (IL6) is a pleiotropic cytokine with either pro- or anti-inflammatory effects depending on the cellular context and the pathophysiological state. IL6 plays a central role in local and systemic manifestations of RA and represents a successful therapeutic target [1, 2]. Stromal and immune cells produce IL6 upon induction with major inflammatory activators such as tumour necrosis factor α (TNFα) or Interleukin 1-beta (IL1β). Normal physiological concentrations of IL6 in human serum are relatively low, but rapidly increase under pathological conditions [3, 4]. Synovial fibroblasts (SF) of the lining are the primary source of IL6 mRNA and protein in the synovium of RA patients [5].

Central to the IL6 context dependent function is the receptor system gp130/IL6R. In contrast to the ubiquitously expressed subunit gp130, the membrane subunit IL6R is mostly restricted to hepatocytes, myeloid cells and some lymphocytes. In contrast resident cells, including fibroblasts, can only respond to IL6 through trans-signaling mediated by soluble IL6 receptor (sIL6R) [6, 7]. While both pro- and anti-inflammatory effects have been associated to signal activation of the fully-competent receptor gp130/IL6R, signaling mediated through gp130/sIL6R has been mostly linked to the induction of pro-inflammatory programs [8]. Interestingly, in the context of inflammatory arthritis, increased synovial sIL6R levels correlate with enhanced joint destruction and leukocyte recruitment [3, 7, 9], but the role of the IL6/sIL6R trans-signaling on inflamed synovial tissue is poorly known.

SF are essential players in RA pathophysiology, undergoing significant hyperplasia in rheumatoid arthritis (RA) and responding to exogenous proinflammatory stimuli, particularly to TNFα, by producing a large variety of proinflammatory, bone destructive and cartilage-destructive mediators [10] thus contributing to perpetuate the inflammatory milieu in the joint. Interestingly, a recent investigation has demonstrated the cooperative role of TNFα and IL6/sIL6R in regulating the cell cycle and viability of synovial fibroblasts cells, accelerating RASF proliferation [11], suggesting that the crosstalk between these two factors may enhance the pathological impact of SF cells in RA.

The hierarchy of TNFα in SF inflammatory activity has been intensively studied in the last decades [12, 13]. SF have a central role in the recruitment and retention of leukocytes in the inflamed joint [14–16] as well as in many other effector mechanisms resulting in chronic inflammation and joint destruction [10].

The clinical efficacy of both TNFα and IL6 antagonists has been extensively demonstrated in RA patients [17]. However, although many efforts have focused in understanding the specific pathogenic role of IL6 in adaptive immunity in arthritis, much less is known about the direct effects of IL6 trans-signaling on the synovial inflammatory process.

In this study, we show that IL6 trans-signaling induces an inflammatory response in SF that modulates the robust TNFα-induced inflammatory signal, by regulating the cytokine and chemokine expression pattern. Furthermore, although IL6/sIL6R has a limited impact on the expression of matrix degrading enzymes, it regulates the expression profile of specific matrix metalloproteases induced by TNFα. Our results point out to a highly orchestrated response to these key cytokines on the SF effect or response in RA.

## Methods

### Patients and cells

SF cultures were established by explant growth of synovial tissues obtained by arthroscopic knee biopsies from patients without previous joint disease at elective arthroscopy for minor traumatic lesions, or patients with RA at the time of prosthetic replacement surgery. Patients signed a written informed consent, and the study was approved by the Ethics Committee of Hospital 12 de Octubre, Madrid, Spain (N° CEI:17/085). All methods involving humans were performed in accordance with the relevant guidelines and regulations. SF were cultured in Dulbecco’s modified Eagle’s medium (DMEM) supplemented with 10% heat inactivated fetal bovine serum (FBS) (Lonza, Verviers, Belgium) and used after 3rd passage. For all tests SF lines were stimulated with either TNFα, IL6/sIL6R, or both together in DMEM 0.5% FBS, the time and dose of the treatment will be indicated for each experiment. TNFα, IL6 and sIL6R (PreproTech, Rocky Hill, NJ, USA) were reconstituted according to manufacturer instructions. Where indicated, cells were treated with inhibitors Actinomycin D (10 μg/ml) (Sigma-Aldrich Quimica SA, Madrid, Spain), Adalimumab (10 μM) (AbbVie, North Chicago, IL, USA), Ruxolitinib (1 μM) (Selleckchem, Houston, TX, USA) and Cycloheximide (5 μM) (Sigma-Aldrich Quimica SA).

### mRNA analysis

First-strand cDNA synthesis was performed using 2 μg of total RNA with High-Capacity cDNA Reverse Transcription kit (Applied Biosystems, Foster City, CA, USA) according to manufacturer protocol. Samples were analyzed by quantitative RT-PCR (RT-qPCR) with gene-specific primer pairs (Additional file 1: Table S1) on an Applied Biosystem 7500 Fast Real-Time PCR System (Applied Biosystem) using Power Sybr Green PCR Master Mix (Applied Biosystems). Values were normalized to those of the endogenous reference (*HPRT1* gene) using the 2^-∆∆Ct^ method. In each case, multiple reactions were performed using 4–6 independent biological replicates.

### Enzyme-linked immunosorbent assay (ELISA)

Concentrations of IL8/CXCL8 and CCL8 in culture supernatants were determined by ELISA (Biolegend Inc., San Diego, CA, USA) according to the manufacturer protocols. The read-out for all ELISAs was carried out with a MultiskanEX plate reader (ThermoScientific).

### Cell migration assay

Mononuclear and polymorphonuclear leukocytes were obtained from peripheral blood from healthy donors (*n* = 7) by density gradient centrifugation using Lympholyte-poly (Cederlane Laboratories, Burlington, Canada). Cell migration was assessed using 6.5 mm Transwell with 5.0 μm pore polycarbonate membrane insert (Corning Inc., Corning, NY, USA). 0.3 × 10^6^ cells in DMEM 0.5% FBS were seeded in the upper chamber of the transwell. In the lower chamber we added conditioned medium from SF cultures (*n* = 4) stimulated for 48 h with either TNFα (10 ng/ml) or IL6 and sIL6R (50 ng/ml each). After an incubation for 4 h, migrated cells from the lower chamber were collected and analyzed by flow cytometry with a BD FACSCalibur instrument (Becton Dickinson, San José, CA, USA). Subpopulations of polymorphonuclear (PMN) and mononuclear cells (MNC) were identified by forward and side scatter. By combining donors and SF cultures, a total of 19 experiments were performed.

### Statistical analysis

Data were analyzed using GraphPad Prism software v6.0 (GraphPad Software, San Diego, CA, USA). Statistical analyses were performed as indicated in the corresponding figure legends. A *p*-value < 0.05 was considered statistically significant (*p < 0.05, **p < 0.01, ***p < 0.001, ****p < 0.0001).

## Results

### Comparative analysis of genes regulated by TNFα or IL6/sIL6R signaling in SF

To investigate the relative contribution of IL6 to the SF inflammatory response, we analyzed the expression pattern of a large group of cytokines, chemokines and matrix metalloproteases (MMPs) with important roles in RA pathophysiology. We first confirmed the lack of effect of IL6 alone compared to IL6/sIL6R or TNFα on the expression of known target genes such as *CCL2* and *IL6* (data not shown) in cultured SF. Furthermore, despite individual baseline differences on gene expression, both RA and non-RA cultured SF respond similarly to TNFα and/or IL6/sIL6R stimulation (data not shown) and therefore, SF from both healthy and RA donors were indistinctly used.

**Fig. 1:**
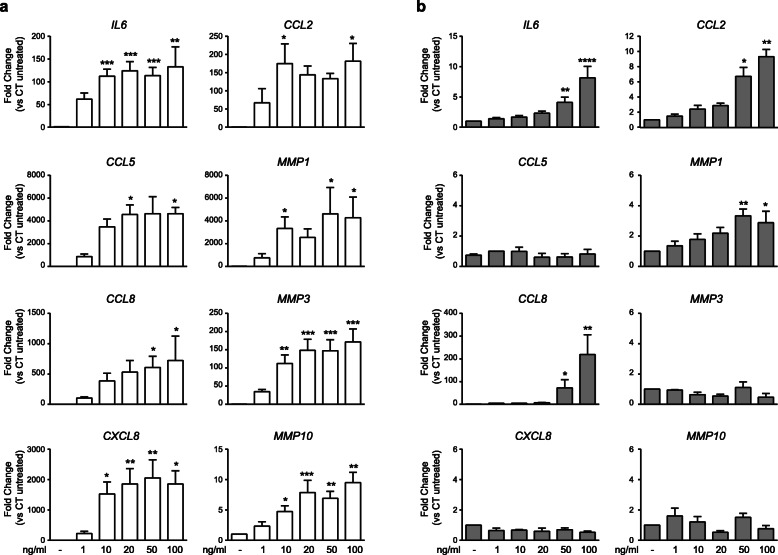
Dose-response expression of genes in SF. SF were stimulated for 24 h with increasing doses of either TNFα (**a**) or IL6/sIL6R (**b**). Graphics show the changes in mRNA expression of indicated genes in relation to untreated control. Mean ± SEM from three to six SF lines (*Kruskal-Wallis with Dunn’s multiple comparisons test)

In dose-response experiments, RT-qPCR analyses showed that TNFα induced the expression of the cytokine *IL6*, chemokines genes *IL8/CXCL8, CCL2, CCL5* and *CCL8*, and MMPs genes *MMP1*, *MMP3* and *MMP10* (Fig. 1a). IL6/sIL6R induced the expression of *IL6* itself, mononuclear-cells recruiting chemokines such as *CCL2 or CCL8* but no the neutrophil-recruiting chemokine gene *IL8/CXCL8* and in contrast to the robust activation of MMPs mediated by TNFα, only *MMP1* was partially expressed upon stimulation by IL6/sIL6R trans-signaling at the higher dose tested (Fig. 1b). The magnitude of gene inductions by IL6/sIL6R was 2–100 times lower than by TNFα.

Overall, IL6 trans-signaling mediates effects partially overlapping to those of TNFα in SF, supporting the coordinated expression of cytokines, chemokines and matrix metalloproteases central to RA pathophysiology.

### IL6/sIL6R trans-signal modulates the TNFα-induced inflammatory response of SF

**Fig. 2:**
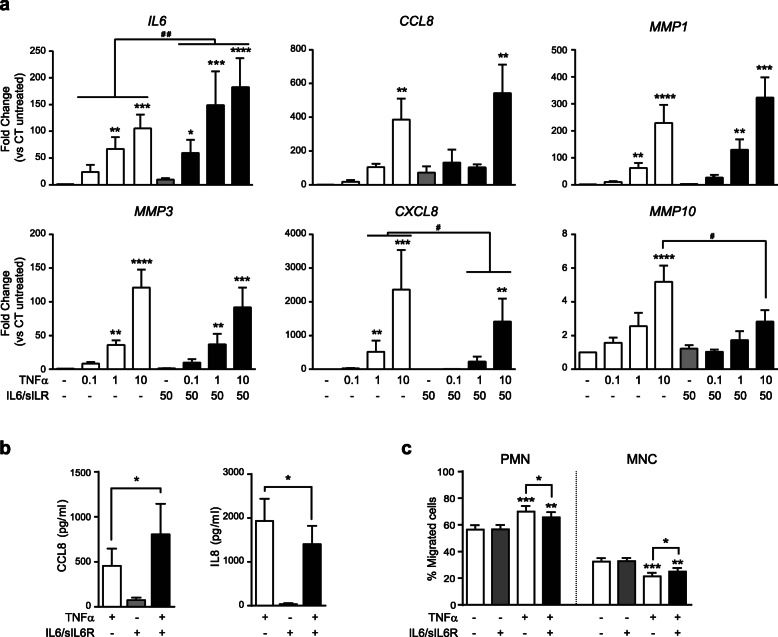
Modulation of TNFα-induced genes by IL6/sIL6R in SF. **a** Change in mRNA expression upon stimulation with increasing doses of TNFα and a fix dose of IL6/sIL6R (50 ng/ml each) in relation to untreated control. Mean ± SEM from three to eight SF lines (*Kruskal-Wallis with Dunn’s multiple comparisons test; ^#^Wilcoxon test). **b** Protein levels of CCL8 and IL8/CXCL8 upon induction with TNFα (10 ng/ml) and IL6/sIL6R (50 ng/ml each). Mean ± SEM from seven SF lines (*Wilcoxon test). **c** Percentage of polymorphonuclear and mononuclear leukocytes in cell migration assays (*n* = 19) (*One-way ANOVA with Tukey’s multiple comparison test)

To investigate IL6 trans-signaling regulation of the inflammatory response in SF, we stimulated SF cultures with different suboptimal doses of TNFα plus a fixed dose of IL6 and sIL6R (50 ng/ml) according to dose-response assays of representative genes regulated by each factor (Fig. 1). Cooperative stimulation of SF with both TNFα and IL6/sIL6R enhanced the expression of a common regulated gene such as IL6 (Fig. 2a). Although the observed increased expression of *CCL8* and *MMP1* was not statistically significant (*p* = 0.25 and *p* = 0.43 respectively, at the higher dose of TNFα), further mRNA and protein expression analysis along the present study demonstrated the consistency of the increase shown for those genes. In contrast, *MMP3*, a gene specifically activated by TNFα but not IL6/sIL6R, was not affected by IL6 trans-signaling (Fig. 2a). IL6 has been shown to regulate the expression pattern of chemokines on stromal cells to drive the transition from the recruitment of neutrophils to mononuclear cells [12]. Consistently, TNFα-induced mRNA and protein expression of the neutrophil-recruiting chemokine IL8/CXCL8 was partially inhibited by the trans-signal activation of IL6/sIL6R in SF, whereas TNFα and IL6/sIL6R cooperated to up-regulate the protein expression of mononuclear leukocytes chemoattractant chemokine CCL8 (Fig. 2a and b). Interestingly, IL6/sIL6R also inhibited the TNFα-induced expression of *MMP10*, an enzyme linked to the resolution of inflammation by macrophages [18] (Fig. 2a).

To analyze the functional implications of the switch in the pattern of chemokines after the cooperative stimulation with TNFα and IL6/sIL6R, we performed a cell migration experiment using conditioned media from SF cultures as chemoattractant for leukocytes. Conditioned media from SF cultures treated with both TNFα and IL6/sIL6R reduced significantly the percentage of polymorphonuclear (PMN) cells in comparison with medium from TNFα-stimulated cultures (Fig. 2c). Likewise, TNFα plus IL6/sIL6R conditioned media induced a significant increase in the recruitment of mononuclear cells (MNC) (Fig. 2c).

### IL6/sIL6R regulates the kinetics of the TNFα-inflammatory response

Expression of genes activated by continuous exposure to TNFα is determined by transcriptional and post-transcriptional mechanisms that regulate its level and kinetics [19–21]. To distinguish the potential regulation of these mechanisms upon induction with IL6/sIL6R, we first set the temporal pattern of induction for analyzed genes.

The kinetics of genes stimulated by TNFα in SF mostly fit into the three broad classes, as previously described in other cell types [19] (Additional file 2: Figure S1a). Thus, the expression of an early gene (*IL6)* was consistently detected at 0.5 h upon induction, while intermediate genes (*CCL2*, *IL8/CXCL8*) are observed before 2 h and late expression genes (*CCL8*, *MMP1*) later than 2 h. The expression of genes mediated by continuous exposure of IL6/sIL6R fit into a similar pattern of induction, although some of the genes that are common to both factors fall into a different category. The induction of *IL6* and *MMP1* follows identical kinetics for both inflammatory factors. In contrast, the expression of *CCL8* or *CCL2* induced by IL6/sIL6R showed faster kinetics than that mediated by TNFα, showing *CCL2* a less stable induction (Additional file 2: Figure S1b). These results more likely reflect differences in the underlying regulatory mechanisms induced by either inflammatory cytokine.

**Fig. 3:**
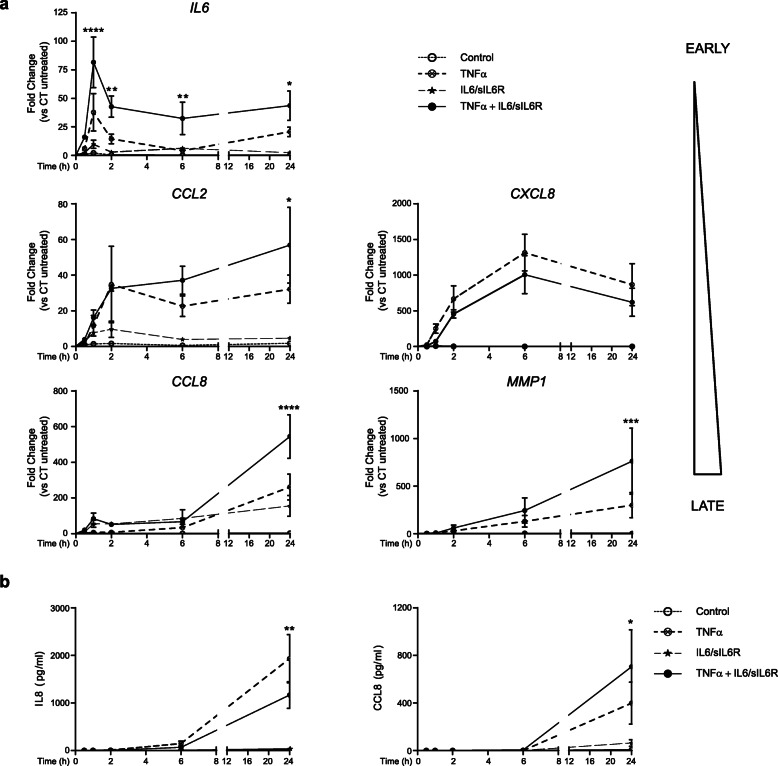
Kinetic patterns of cooperative gene expression in SF. SF were cultured in the presence or absence of TNFα (10 ng/ml), IL6/sIL6R (50 ng/ml each) or both. **a** Representative genes *IL6*, *CCL2*, *IL8/CXCL8*, *CCL8*, and *MMP1* mRNA expression of four to six independent cultures. **b** IL8/CXCL8 and CCL8 protein in supernatants of six independent SF cultures. Values are mean ± SEM (vs control, *2way ANOVA with Fisher’s LSD multiple comparisons test)

The expression kinetics of genes co-stimulated with TNFα and IL6/sIL6R may provide information about the molecular mechanisms operating in the cooperative induction of genes. For all TNFα-induced genes, kinetics was maintained after co-stimulation with IL6/sIL6R, but differences were observed in the time of the cooperative effect (Fig. 3). The increase of *IL6* and the decrease of *IL8/CXCL8* expression by IL6/sIL6R was detectable as soon as 30 min upon induction, more likely showing changes in transcription and/or mRNA stability mechanisms. However, increased expression of intermediate and late expression genes such as *CCL2*, *CCL8* or *MMP1* occurs at later time, suggesting that secondary factors may underlie the cooperative expression of these genes (Fig. 3a). A similar pattern of expression was obtained when we analyzed the protein released to the culture medium (Fig. 3b). Although not statistically significant, a moderate IL8/CXCL8 inhibition was detectable at 6 h after induction, while enhanced expression of CCL8 was only detectable later at 24 h (Fig. 3b).

### IL6/sIL6R modulates the inflammatory expression profile through de novo transcriptional mechanisms

**Fig. 4:**
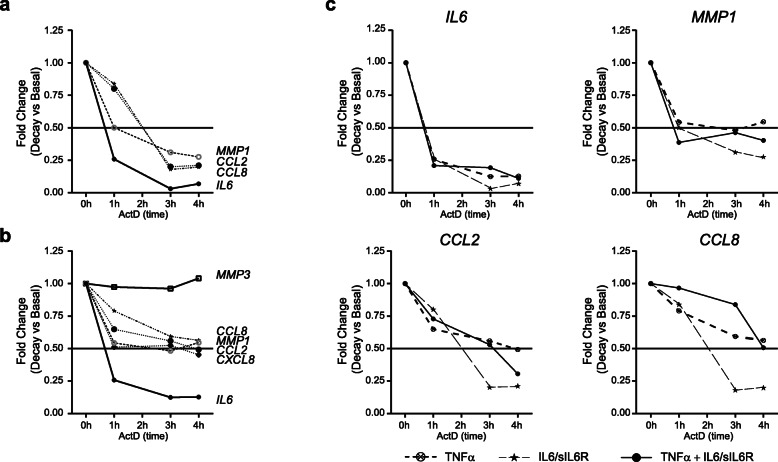
Stability of mRNA encoded by early and late genes. SF were stimulated for 24 h with either IL6/sIL6R (50 ng/ml each) (**a**), TNFα (10 ng/ml) (**b**), or both (**c**). Change of *IL6*, *CCL2*, *IL8/CXCL8*, *CCL8*, *MMP1* and *MMP3* mRNA expression are relative to the baseline value before the addition of actinomycin D (ActD) (0 h). Data are the mean of three independent experiments

Accumulation of mRNA may be influenced by ongoing transcription or mRNA stability. To investigate the relative contribution of mRNA stability to the induction of genes mediated by TNFα or IL6/sIL6R in SF, we measured changes in mRNA expression over time after blocking transcription with actinomycin D (ActD). We determined mRNA expression after 24 h of induction with either TNFα or IL6/sIL6R, relative to the baseline value before the addition of ActD. Our results showed that all tested genes stimulated by IL6/sIL6R responded similarly, with half-lives of mRNA transcripts decay varying from 0.8 to 2 h (Fig. 4a). In contrast, mRNAs induced by TNFα were on average more stable, lasting more than 2 h for most genes. The half-life of decay for *IL6* mRNA stimulated by TNFα was shorter than for the rest of the genes, but similar to that stimulated by IL6/sIL6R (0.8 to 2 h) (Fig. 4b). We could not determine the half-life of decay for *MMP3*, since we found no consistent decrease in stability up to 4 h after treatment with ActD. These results suggest that, while both transcriptional and post-transcriptional mechanisms are involved in the modulation of TNFα induced genes, the short half-life of IL6/sIL6R induced mRNAs may reflect a dominant role for de novo transcription. We also observed that co-stimulation of SF with both TNFα and IL6/sIL6R did not significantly modify the half-life of analyzed genes (Fig. 4c), suggesting that regulation of the mRNA stability of TNFα-induced genes is not affected by the modulation after trans-signal activation of IL6/sIL6R.

**Fig. 5:**
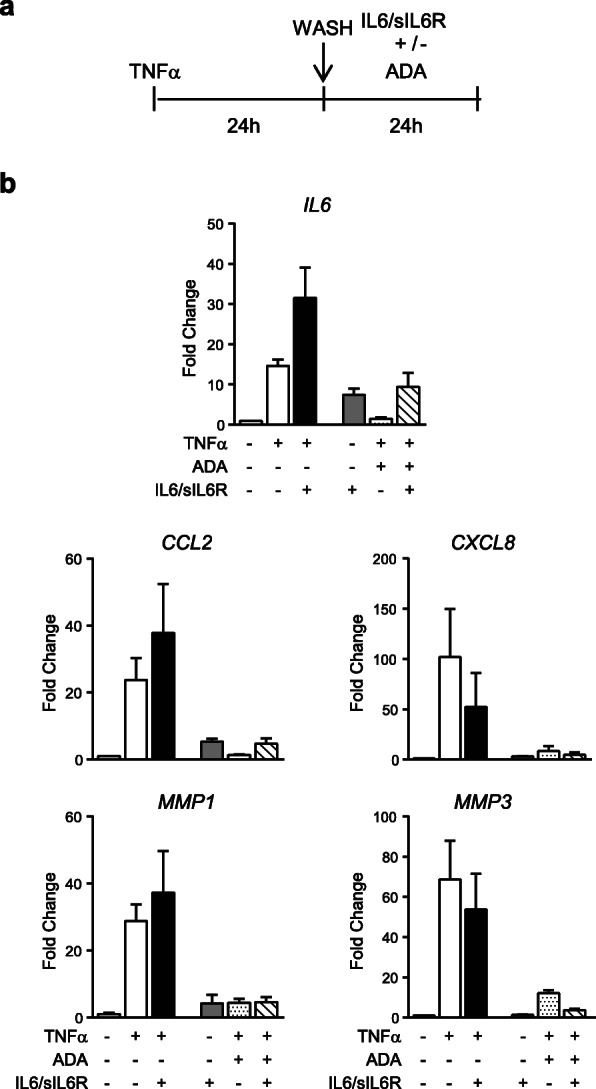
Effect of adalimumab on TNFα-induced genes modulated by IL6/sIL6R. **a** Schematic representation of the experiment. SF were stimulated with TNFα (10 ng/ml) for 24 h. Inflammatory input was removed by washing cells and the second stimuli IL6/sIL6R (50 ng/ml each) was added in the presence of adalimumab (ADA) (10 μg/ml) to block any residual TNFα. **b** Graphics show the change on the mRNA expression of representative genes. Untreated SF was used as reference. Data are mean ± SEM of three independent experiments

Recent reports have also demonstrated that a prolonged TNFα exposure for longer than 24 h promotes the stability of mRNA expression in fibroblasts by epigenetic mechanisms, influencing the temporal order of induction of inflammatory genes [19, 21, 22]. To distinguish the potential role of these priming mechanisms, we first examined gene expression changes in response to TNFα withdrawal. We cultured SF in the presence of TNFα for 24 h, removed the inflammatory input by washing the cells, and added new medium with adalimumab (ADA) to block residual TNFα and with IL6/sIL6R for additional 24 h (Fig. 5). SF pre-exposed to TNFα and treated with ADA did not display enhanced induction of early (*IL6*, *CCL2* and *IL8/CXCL8*) nor late genes (*MMP1* and *MMP3*) after IL6/sIL6R treatment, showing expression levels similar to the induced with only IL6/sIL6R (Fig. 5). We also observed that mRNA expression of *MMP3* was more resistant to TNFα withdrawal probably due to its stability. These data suggest that the observed cooperative effect requires concomitant induction by both TNFα and IL6/sIL6R.

These results collectively support the view that, although regulation of the mRNA stability and priming mechanisms may determine the kinetics of TNFα-induced gene expression, cooperative induction by TNFα and IL6/sIL6R is more likely mediated through coordinated de novo transcriptional mechanisms.

### Late crosstalk between TNFα and IL6/sIL6R is mediated by activation of the JAK-STAT pathway

The delayed effect on the cooperative expression of genes such as *CCL8*, *CCL2* or *MMP1* (Fig. 3a) suggested that either TNFα or IL6/sIL6R induces secondary mechanisms dependent of new protein synthesis. This hypothesis was tested by inhibiting the protein synthesis with cycloheximide (CHX). CHX inhibited the expression of late genes such as *CCL8* and *MMP1* induced by either TNFα (Additional file 3: Figure S2a) or IL6/sIL6R (Additional file 3: Figure S2b), suggesting that protein-synthesis-dependent pathways are partly involved in the expression of these genes in SF, in contrast to early or intermediate genes such as *IL6, IL8/CXCL8* and *CCL2.*

**Fig. 6:**
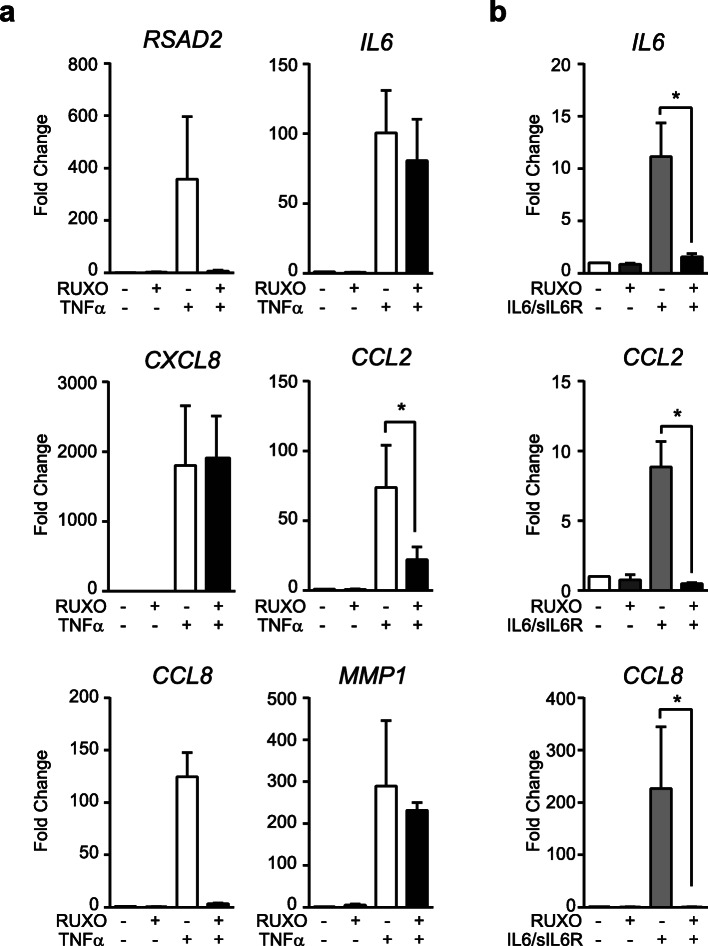
Inhibition of TNFα-induced genes by ruxolitinib in SF. TNFα-induced (10 ng/ml) (**a**) and IL6/sIL6R-induced (50 ng/ml each) (**b**) mRNA expression for 24 h in the presence and absence of ruxolitinib (RUXO) (5 μg/ml). Results are mean ± SEM from three to six independent SF cultures (*Wilcoxon test)

Previous investigations have demonstrated that TNFα stimulation of SF induces the expression of several lymphocyte-attracting chemokines through a JAK signaling-mediated mechanism, dependent on autocrine release of type I Interferons (IFN) [23]. To confirm this possibility in our model, we analyzed the *RSAD2* mRNA expression, a classical IFN-induced gene, after TNFα treatment. We observed a high induction of *RSAD2* that was completely inhibited in the presence of the JAK/STAT inhibitor ruxolitinib (RUXO) (Fig. 6a). As expected, induction of IL6/sIL6R dependent genes was abrogated by RUXO treatment (Fig. 6b). Further analyses demonstrated that RUXO significantly inhibited TNFα-induced expression of *CCL2* and *CCL8*, implying that the secondary mediator acts through JAK/STAT. Interestingly, *MMP1,* other late gene regulated by CHX was not affected by RUXO, suggesting that a JAK/STAT-independent but protein-synthesis-dependent pathways is partly involved in *MMP1* expression.

These experiments further revealed that a TNFα-induced autocrine mechanism is regulating part of the TNFα expression program in SF. This autocrine mechanism induced by TNFα shares a common JAK/STAT signaling pathway with IL6/sIL6R that may partially account for the cooperative expression of specific genes.

## Discussion

The response of resident cells in the synovium is essential to RA pathogenesis and it is conditioned by inflammatory cytokines. Although, the function of the two pivotal cytokines, TNFα and IL6, has been extensively studied in this context, most investigations have focused on the role of TNFα as the main upstream inductor of IL6 expression. Our present work shows that IL6/sIL6R signaling is able to modulate the TNFα inflammatory response elicited in SF, promoting changes of the inflammatory gene expression pattern associated to RA pathogenesis. Furthermore, we provide insight into the molecular mechanisms that regulate the crosstalk between both cytokines.

The mode of action of IL6 is contextual, defined by other factors present within the local milieu [10]. Although IL6 is defined as an inflammatory cytokine, it does not directly induce leukocyte recruitment [15]. Rather, previous studies have shown the ability of IL6, either alone or cooperating with other factors, to modify inflammatory infiltrates [24–28]. Analysis of adjuvant-induced arthritis(AIA) in IL6^+/+^ and IL6^−/−^ mice demonstrated that IL6 deficiency is associated with reduced synovial infiltration and is accompanied by a defect in both CCL2 expression and the recruitment of leukocytes bearing the CCL2 receptor CCR2 [6]. Our results show that the induction of IL6/sIL6R signaling in SF mainly stimulates the expression of cytokines and chemokines that attract lymphocyte and monocyte cells, although at a lower level than TNFα. However, the cooperative activation of both factors further enhances the expression of common chemokines and cytokines, thus potentially contributing to sustain the leukocyte influx into the synovial tissue. The impact of IL6/sIL6R and TNFα combination in SF may be more relevant in the context of RA where the local increase of sIL6R correlates with the extent of leukocyte infiltration and joint destruction [3, 9]. Because the expression of IL6R within the RA synovial environment is mainly restricted to leukocytes [29], infiltrating cells may be the source of the synovial sIL6R in response to inflammatory mediators such as TNFα [26, 30–32]. The sIL6R would then be acting as a paracrine mediator on synovial cells, further inducing leukocyte recruitment during inflammation [33]. Several studies have highlighted a role for sIL6R in regulating the expression of chemokines and adhesion molecules [8, 31, 32, 34–37], thus mediating the transition between the early neutrophilic stage of acute inflammation and the more sustained mononuclear cell influx [26]. Interestingly, mouse models of IL6^−/−^ showed reduced neutrophil accumulation at sites of infection or inflammation that seems to be secondary to the effects of IL6 trans-signaling on stromal cells [38]. Our studies demonstrate that IL6/sIL6R shapes the expression program of leukocyte recruiting factors mediated by TNFα in SF. In addition, these results confirm that IL6 trans-signaling directly enhanced TNFα-induced expression of monocyte- and lymphocyte-regulating factors such as IL6, CCL2, and CCL8, while inhibiting the expression of the neutrophil-recruiting factor IL8/CXCL8. The functional relevance of these changes in gene expression patterns are highlighted by the increased MNC migration at the expense of PMN recruitment upon IL6/sIL6R trans-signaling. Such scenario may be relevant to arthritis, in which neutrophils may play an essential role in the initiation of RA but mononuclear leukocytes infiltration would explain chronicity [39, 40]. In this context, regulation of the SF inflammatory response by IL6/sIL6R would be playing a major role in the transition to sustained inflammation.

The temporal order of induction and the relative duration of the various inflammatory events induced by TNFα and IL6/sIL6R suggests a gene activation program that ensures a rapid inflammatory response [19–21]. Kinetics and stability analyses of gene expression, as well as the differential decay for each gene induced by TNFα suggest that, similar to what is described in other cells types [19], SF display a pattern of gene expression controlled by both transcriptional and post-transcriptional mechanisms. In contrast, the fast decay observed in IL6/sIL6R-induced mRNAs suggests that de novo transcriptional mechanisms are mainly, if not exclusively, regulating the expression genes induced by IL6/sIL6R in SF, even for the stable expression of genes such as *CCL8* or *MMP1*. Furthermore, IL6/sIL6R signaling did not modify the stability of TNFα-expressed mRNAs, suggesting an independent transcriptional regulation of commonly induced genes.

Evidences suggest that chronic pathological states are associated with disease-specific stable changes in gene expression, many of them consistent with epigenetic mechanisms. It has been shown that TNFα can display a gene-specific priming effect on RASF, mediated by epigenetic changes, that enhances subsequent inflammatory response to other factors such as IFNs [22]. However, our data do not demonstrate TNFα priming of gene expression induced by IL6/sIL6R. Altogether, our results suggest that the cooperative effect elicited by IL6/sIL6R in TNFα-responses requires the sustained presence of both factors, potentially making SF more responsive to the pharmacological intervention with IL6/sIL6R signaling inhibitors.

IL6/sIL6R activation of SF may not only contribute to synovitis by sustaining leukocyte infiltration within the inflamed joint, but also enhancing their matrix degrading activity. Similar to previous investigations, we observed a consistent up-regulation of *MMP1* expression upon IL6/sIL6R stimulation of SF [41]. For most of the MMPs, TNFα induces activation via several transcription factors, including NF-kB and activator protein 1 (AP-1) [42]. In contrast, IL6/sIL6R effect on these genes seems to be more specific. For instance, *MMP1* and *MMP3* genes bear STAT binding sites [43] that would make them susceptible of IL6-induction by promoter-bound STAT3 or STAT1, in contrast to other MMPs gene promoters that do not have STAT motifs [44]. Although differences in the cell sources used in both studies may account for part of the discrepancies between Araki’s results [41] and ours, the fact that *MMP3* is not expressed at any dose tested, even under active transcriptional conditions such as those imposed by TNFα, suggests that IL6 does not induce the recruitment of transcription factors to the *MMP3* promoter, further supporting that de novo transcription has a major role in the response mediated by IL6/sIL6R signaling.

Of interest is the IL6/sIL6R inhibitory effect on TNFα-mediated expression of *MMP10.* MMP10 is linked to the control of the resolution phase of inflammation in models of pulmonary inflammation and experimental colitis, where MMP10 deficiency exacerbates the disease [18, 45, 46]. Therefore, IL6/sIL6R may also potentiate TNFα inflammatory response by restraining this anti-inflammatory loop.

## Conclusions

Although our study mostly rely on mRNA expression analysis, thus limiting the interpretation of our conclusions at the pathological level, our findings places SF as a relevant target for IL6 trans-signaling response that contributes to leukocyte infiltration and joint destruction through direct effects and by modulating TNFα actions (Additional file 4: Figure S3), explaining the local effects of IL6-targeted DMARDs with independence of their immunoregulatory potential.

## Supplementary information

**Additional file 1: Table S1.** Primer sequences used for quantitative real-time PCR analysis.

**Additional file 2: Figure S1**. Kinetic patterns of gene expression in SF. SF were cultured in time-course experiments in the presence or absence of TNFα (10 ng/ml) (a), or IL6/sIL6R (50 ng/ml each) (b). An extended analysis of genes was measured by RT-qPCR, in addition to genes from Fig. 3 also depicted here. Data are mean ± SEM from three to six independent cultures.

**Additional file 3: Figure S2**. Effect of cycloheximide on TNFα- and IL6/sIL6R-induced genes. TNFα-induced (10 ng/ml) (a) and, IL6/sIL6R-induced (50 ng/ml each) (b) mRNA expression for 24 h in the presence and absence of cycloheximide (CHX) (10 μM). Mean ± SEM from three to six independent SF cultures.

**Additional file 4: Figure S3**. Scheme of the cooperative role of TNFα and IL6/sIL6R in regulating the inflammatory response in SF. (a) TNFα induces a strong inflammatory response in SF during RA, mediating the infiltration of monocytic and leukocytic cells as well as neutrophils, the expression of matrix degradative metalloproteases, but also potentially activating mechanisms to control the inflammatory program. Part of these effects are mediated through activation of JAK/STAT signaling pathways. (b) In this TNFα- inflammatory context, IL6/sIL6R would be playing a major role in the transition to sustained inflammation, enhancing leukocyte infiltration and joint destruction.

## Data Availability

The datasets analyzed in the current study are available from the corresponding author on reasonable request.

## Abbreviations

ActD: Actinomycin D
ADA: Adalimumab
CHX: Cycloheximide
DMARDs: Disease-modifying antirheumatic drugs
DMEM: Dulbecco’s modified Eagle’s medium
ELISA: Enzyme-linked immunosorbent assay
FBS: Fetal bovine serum
IFN: Interferon
IL6: Interleukin-6
MMP: Matrix metalloprotease
MNC: Mononuclear cells
PMN: Polymorphonuclear
RA: Rheumatoid arthritis
RASF: Rheumatoid Arthritis Synovial Fibroblasts
RT-qPCR: Quantitative RT-PCR
RUXO: Ruxolitinib
SF: Synovial fibroblasts
sIL6R: Soluble IL6 receptor
