# Supplementary material for: IL6/sIL6R regulates TNFα-inflammatory response in synovial fibroblasts through modulation of transcriptional and post-transcriptional mechanisms
Source: BMC Mol Cell Biol. 2020 Oct 30;21:74. doi: 10.1186/s12860-020-00317-7 (PMC7596982; doi:10.1186/s12860-020-00317-7)
Supplement: Supplementary file 1 — Additional file 1: Table S1. Primer sequences used for quantitative real-time PCR analysis. [file 12860_2020_317_MOESM1_ESM.pdf]

Table S1. Primer sequences used for quantitative real-time PCR analysis

Gene Name	Forward Primer Sequence	Reverse Primer Sequence
IL6	5'- GTGGCTGCAGGACATGACAA -3'	5'- TGAGGTGCCCATGCTACATTT -3'
CXCL8	5’- AAGAGCCAGGAAGAAACCACC -3’	5’- CTGCAGAAATCAGGAAGGCTG -3’
MMP3	5'- CTGCTGTTGAGAAAGCTCTG -3'	5'- AATTGGTCCCTGTTGTATCCT -3'
CCL2	5’- ACTCTCGCCTCCAGCATGAA -3’	5’- TTGATTGCATCTGGCTGAGC -3’
CCL5	5'- CTCCCCATATTCCTCGGACA -3'	5'- GTTGATGTACTCCCGAACCC -3'
IL7	5'- CAACACAGACTCGGCAACTC -3'	5'- CAGAACAAGGATCAGGGGAGG -3'
CCL8	5'- GTTTCTGCAGCGCTTCTGTG -3'	5'- GTTTCTGCAGCGCTTCTGTG -3'
MMP10	5'- TCTGAGATGCCAGCCAAGTG -3'	5'- GGGTTCCAGTGGGATCTTCG -3'
RSAD2	5'- GCTTCTGTTTCCACACAGCC -3'	5'- CAGCCGCAACTCTACTTTGC -3'
ADAMTS4	5'- CATGTGCAACGTCAAGGCTC -3'	5'- TCTTGTCATCTGCCACCACC -3'
MMP1	5'- TGGACGTTCCCAAAATCCTG -3'	5'- AAGGGATTTGTGCGCATGTAG -3'
SOCS3	5’- GACGGAGACTTCGATTCGGG -3’	5’- GGGAAACTTGCTGTGGGTGA -3’
IL26	5’- AGCATGGCTCAAAGCAACG -3’	5’- CCATGAAGAAGGACAGAAGCTG -3’
PLA2G2A	5’- AGTTTAGCAACTCGGGGAGC -3’	5’- GGAAGAGGGGACTCAGCAAC -3’
ADAMTS16	5’- CGGACTGGTCTTCTTGGTCC -3’	5’-GAGCCCTCACAGAACTTCCC -3’
HPRT1	5'- GCTGAGGATTTGGAAAGGGTG -3'	5'- CAGAGGGCTACAATGTGATGGC -3'
