# Supplementary figures and images for: IL6/sIL6R regulates TNFα-inflammatory response in synovial fibroblasts through modulation of transcriptional and post-transcriptional mechanisms
Source: BMC Mol Cell Biol. 2020 Oct 30;21:74. doi: 10.1186/s12860-020-00317-7 (PMC7596982; doi:10.1186/s12860-020-00317-7)

Figure S1

a

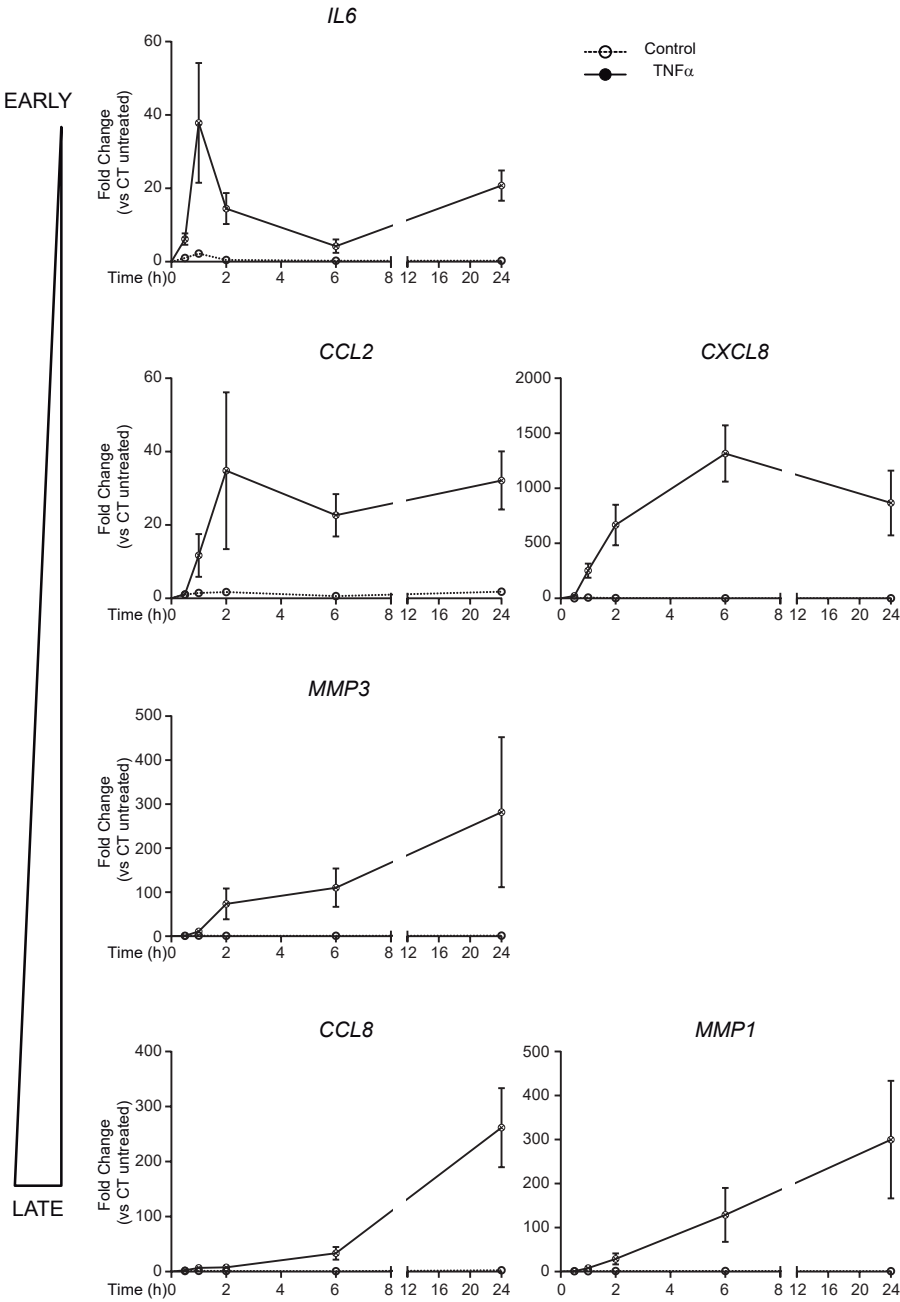

b

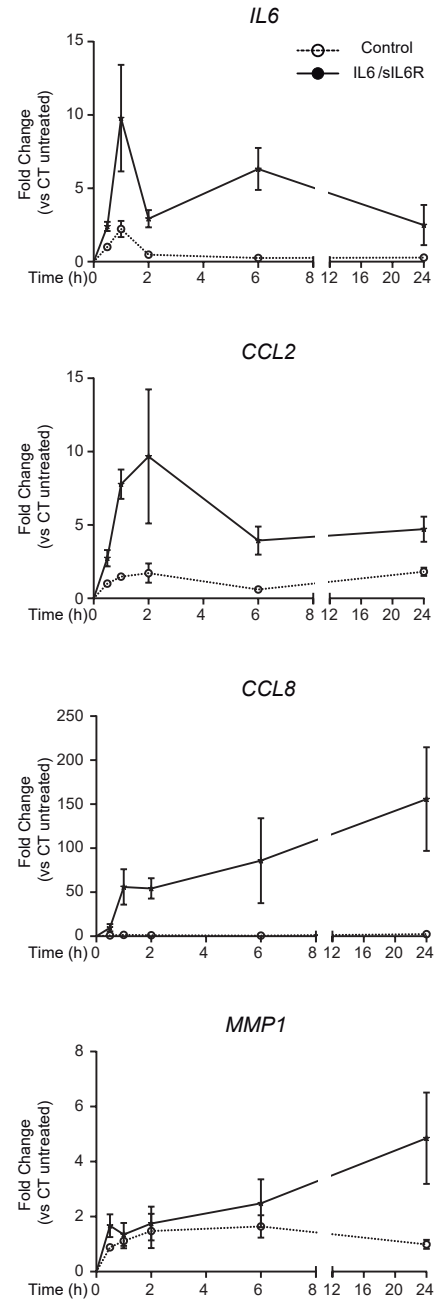

Supplement: Supplementary file 2 — Additional file 2: Figure S1. Kinetic patterns of gene expression in SF. SF were cultured in time-course experiments in the presence or absence of TNFα (10 ng/ml) (a), or IL6/sIL6R (50 ng/ml each) (b). An extended analysis of genes was measured by RT-qPCR, in addition to genes from Fig. 3 also depicted here. Data are mean ± SEM from three to six independent cultures. [file 12860_2020_317_MOESM2_ESM.pdf]

Figure S2

a

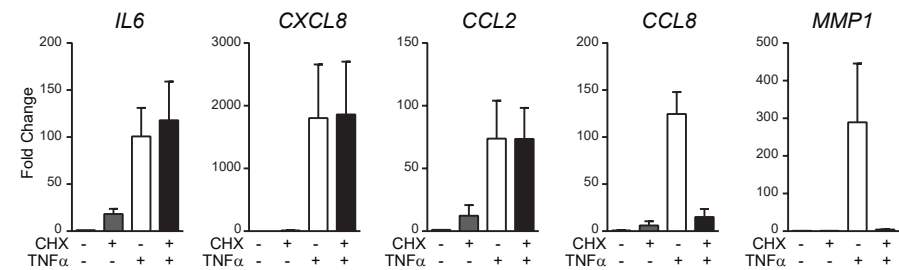

b

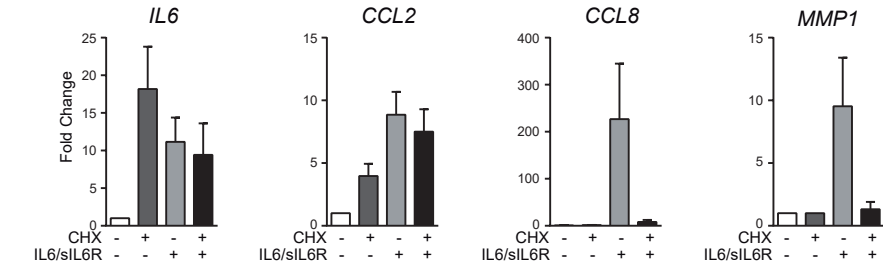

Supplement: Supplementary file 3 — Additional file 3: Figure S2. Effect of cycloheximide on TNFα- and IL6/sIL6R-induced genes. TNFα-induced (10 ng/ml) (a) and, IL6/sIL6R-induced (50 ng/ml each) (b) mRNA expression for 24 h in the presence and absence of cycloheximide (CHX) (10 μM). Mean ± SEM from three to six independent SF cultures. [file 12860_2020_317_MOESM3_ESM.pdf]

Figure S3

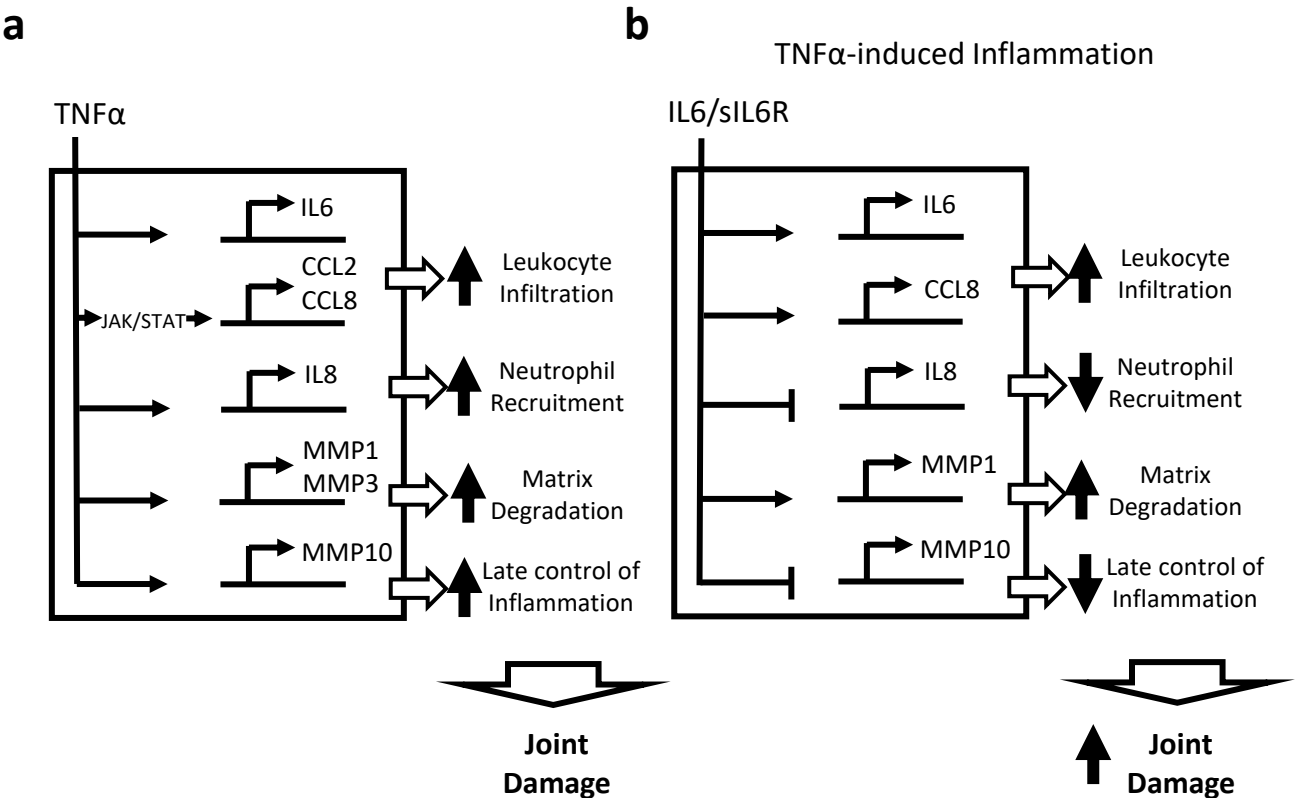

Supplement: Supplementary file 4 — Additional file 4: Figure S3. Scheme of the cooperative role of TNFα and IL6/sIL6R in regulating the inflammatory response in SF. (a) TNFα induces a strong inflammatory response in SF during RA, mediating the infiltration of monocytic and leukocytic cells as well as neutrophils, the expression of matrix degradative metalloproteases, but also potentially activating mechanisms to control the inflammatory program. Part of these effects are mediated through activation of JAK/STAT signaling pathways. (b) In this TNFα- inflammatory context, IL6/sIL6R would be playing a major role in the transition to sustained inflammation, enhancing leukocyte infiltration and joint destruction. [file 12860_2020_317_MOESM4_ESM.pdf]
